# Modeling and optimization of the terminal control area with holding unit

**DOI:** 10.1371/journal.pone.0241204

**Published:** 2021-03-02

**Authors:** Yun-Xiang Han

**Affiliations:** State Key Laboratory of Fundamental Science on Synthetic Vision, Sichuan University, Chengdu, China; Shandong University of Science and Technology, CHINA

## Abstract

Aiming at the problem of resource allocation for departure flights at congested airports, this research explores the optimal configuration of holding units in the terminal control area (TCA). Similar to the job-shop scheduling problem, this problem is solved through a max-plus model with additional constraints. In particular, two optimization models are constructed, in which air segments, departure fixes and holding units are explicitly modeled. Based on the realistic airspace network selected, the proposed models are tested using actual flight data. The experimental results show that the models proposed have better qualities compared with the other methods.

## Introduction

The increasing air traffic flow has led to high congestion in the TCA, such as holding delays for arriving flights and the long queues for departure flights. Typically, TCA is a specific controlled airspace surrounding an airport. Due to the restrictions on investment in airport construction or expansion, aviation authorities are exploring new approaches to increase the utilization rate of existing infrastructure in the TCA [1]. Therefore, the optimal configuration of the holding unit for departure flights becomes a hot topic. In practice, the delay is usually caused by the circling and stacking of aircraft at different flight levels, which is called "holding mode". Once the aircraft enters the holding mode, it must fly according to a specific pre-set mode. In addition, once an aircraft exits the holding mode, it will proceed along the prescribed standard trajectory, and there will be no conflict between flights under normal circumstances.

## Literature review

As we all know, the bottleneck of the airspace network is the capacity of the area around the airport. In other words, the airspace network is highly correlated with the number of arrival/departure flights near the airport, and this is the trigger for the delay propagation. In this case, a heterogeneous network model that considers congestion connections/degrees is applied to predict departure flight delays [2, 3]. In order to alleviate flight delays, it is also necessary to understand the mechanism of air traffic congestion and its propagation process [4, 5]. In this way, the congestion management module can be employed to describe the performance and throughput of the airspace. Today’s methods usually include rerouting or reduction of flight [6, 7].

When choosing the most appropriate actions to alleviate airport congestion, the decision-making must reflect the multi-criteria nature of the problem. Therefore, several challenging results have been proposed for reducing the propagation of perturbations in airspace [8]. In the absence of a substantial increase in airspace capacity, airport congestion can only be alleviated through scheduling intervention or improving capacity utilization [9].

When the airport is crowded or the runway is busy, the aircraft cannot land immediately. At this time, it will join the preset holding mode which make it circling around the airport until the air traffic controller issues a landing signal [10]. Decision support systems based on optimized algorithms can maximize the use of TCA’s available capacity. They will affect the flight time, which is our optimization goal. In the existing literature, the flight scheduling problem is usually regarded as a single-queue optimization problem, from which the aircraft can be pulled out and "paused" [11, 12]. In this context, an optimized scheduling model is designed for uncontrolled airports, and then an optimized scheduling algorithm is tailored for this model [13].

It can be seen from the literature review that recent research mainly focus on improving the operational efficiency of arrival & departure flight. However, none of the existing methods provide sufficient flexibility to assist air traffic controllers in controlling departure flights. It is worth noting that many models ignore the problem of airspace structure optimization related to scheduling, or the configuration of holding units that require decision-making. Consequently, these deficiencies seriously affect the effectiveness of the output as a decision support tool.

Some studies try to overcome these shortcomings by introducing various constraints in the real world, such as landing time windows and priority restrictions. For example, an alternative graph model with additional constraints can be used to solve the aircraft scheduling problem with routing flexibility [14]. Then, a growing number of studies regard this problem as a "job shop" scheduling process [15, 16]. In view of the above discussions, it is necessary to establish a model that is more in line with the realistic situation and incorporate various constraints in air traffic control. Therefore, further research is needed to develop effective algorithms and find the optimal number of configurations for each holding unit.

In addition, dynamic programming (DP) is a method for solving sequential optimization problems consisting of multiple decisions, so that the optimal solution can be obtained from the optimal solution of the sub-problem corresponding to the original problem. In particular, the application of several dynamic programming algorithms in air traffic control is given. For some objective functions, dynamic programming algorithms can be used to obtain the optimal order of flights [17, 18]. Airspace capacity may be reduced due to convective weather, which will cause traffic congestion. Therefore, the optimization of air traffic flow in congested airspace with dynamic convective weather is a challenging problem [19]. In this context, a dynamic programming algorithm is designed to generate a robust solution for airport runway operation [20–22]. More importantly, the stochastic dynamic programming algorithm can well adapt to various operational constraints imposed by TCA, such as the minimum interval requirement between consecutive aircraft, the earliest and latest arrival/departure time of each aircraft, etc. [23, 24].

This paper is organized as follows. Section 3 introduces the formulation of mathematical model, including constraints and objective functions. Section 4 discusses different system configurations and their optimization results. Finally, this article gives a conclusion and proposes further research directions.

## The model and problem definition

For each TCA, the landing aircraft lands on the runway along a predetermined route and standard descent profile from the approach fixes. Similarly, the takeoff aircraft leaves the runway and flies along the ascending profile towards the designated departure fixes. In order to complete the takeoff procedure, each aircraft needs to traverse a certain amount of TCA resources [25].

In addition, most optimization models in the existing literature model TCA as a single resource, these models ignore the air segments in the TCA and are therefore not realistic. More specifically, the aircraft circling in the holding units and the aircraft queued in the departure queue can be represented as "operation" waiting for the air segment to be processed. The processing time of the operation is equal to the time required for the aircraft to traverse a specified resource, depending on the characteristics of the aircraft. This section introduces concrete expressions of decision variables, constraints and objective functions. In the max-plus model given below, an operation refers to the movement of a specific aircraft [26].

According to this representation, the departure route can be subdivided into *N* segments, *M*_1_,*M*_2_,…,*M*_*N*_ with *N*−1 holding units *B*_1_,*B*_2_,…,*B*_*N*−1_ between them. Generally, it takes a certain period of time to transverse a given air segment, which is usually known, because each flight has a scheduled speed sequence. Each holding unit has *S*_*i*_(*i* =1,2,…,*N*), candidate flight levels. Let *x*_*i*_(*j*) and *t*_*ij*_ be the completion time of operation to be performed by flight *i* on the sub-segment *j*, and processing time, respectively. The result is a no-waiting "job shop" scheduling model in which aircraft with given earliest arrival (departure) time is modeled as a "job" with a release date. However, it should be noted that it is necessary to avoid congestion in the fixes for the ‘operations’ [27].

**Definition 3.1 [Non-blocking of departure route]** If the completion time of each flight "processed" by an air-segment is equal to the time instant when the flight departs from the same air-segment, then the air-segment is said to be non-blocking.

**Theorem 3.2 [State equations of air segments with holding units]** For the serial departure routes with *n* sub-segments and *m* flights, the state equation can be described by:

$$x_{i}\left(j+1\right)=t_{j-1,i}⊗\left[x_{i-1}\left(j-1\right)⊕x_{i}\left(j\right)\right]⊕x_{i-1}\left(j-b_{i-1}+1\right)\left(i=1,2,\ldots ,n;j=0,1,\ldots ,m\right)$$ (1)

**Theorem 3.3 [State equations of air segments without holding unit]** For the serial departure routes with *n* sub-segments and *m* flights, the state equations without holding unit can be described by:

$$x_{i}\left(j+1\right)=\left\{ \begin{array}{l} \left[t_{j-1,i}⊗x_{i}\left(j\right)\right]⊕x_{i-1}\left(j\right),\;i=1\\ \left[t_{j-1,i}⊗x_{i-1}\left(j-1\right)\right]⊕x_{i-1}\left(j\right),i\geq 2 \end{array}\right.\left(j=0,1,\ldots ,m\right)$$ (2)

**Theorem 3.4 [State vector equations of air segments with holding units]** For the serial departure routes with *n* sub-segments and *m* flights, let ***x***(*j*) = [*x*_1_(*j*),*x*_2_(*j*),…,*x*_*n*_(*j*)]^*T*^ be the state vector, the vector equation with finite holding unit can be described by:

$$x\left(j+1\right)=T\left(j\right)⊗x\left(j-1\right)⊕E⊗{\left[x_{1}\left(j-b_{1}+1\right),\ldots ,x_{n}\left(j-b_{m-1}+1\right)\right]}^{T}$$ (3)

where ***T*** is a lower triangular matrix and

$$T\left(j+1\right)=\left[\begin{array}{cccc} t_{j,1} & & & \\ {\overset{2}{\underset{l=1}{\Pi }}}_{⊗}t_{j,l} & t_{j,2} & & \\ \vdots & \vdots & \ddots & \\ {\overset{n}{\underset{l=1}{\Pi }}}_{⊗}t_{j,l} & {\overset{n}{\underset{l=2}{\Pi }}}_{⊗}t_{j,l} & \cdots & t_{j,n} \end{array}\right],\;E=[\begin{array}{cccc} \varepsilon & 0 & & \varepsilon \\ & \ddots & \ddots & \\ & & \ddots & 0\\ \varepsilon & & & \varepsilon \end{array}]$$

In particular, when the aircraft traverse the same resource (air segment or holding unit) and the preset minimum safety interval is violated, a potential conflict will occur [28].

**Lemma 3.5 [Sufficient and necessary conditions of non-blocking]** For the serial departure routes with *n* sub-segments and *m* flights, if it is not blocked, the state equations with finite holding units satisfy:

x(j+1)=T(j)⊗x(j)(j=0,1,…,m−1) (4)

In fact, the performance of the holding unit is a key indicator to measure the congestion and stability of the airspace. In an airspace with dense traffic flow, the congestion at a fix usually causes a series of chain reactions upstream, which propagates to the entire airspace. In summary, the optimization problem can be described in detail as follows: Given a set of planned landing/take-off aircraft, and each aircraft’s entry/exit route and their scheduled speed, the optimization model needs to allocate the holding units to each approach/departure route so that all conflicts between aircraft will be solved and the given performance indicators is optimized.

For the limited resources in the TCA, we search the optimal number for each holding unit (*q*_1_,*q*_2_,…,*q*_*k*−1_) which minimizes the total number of holding unit *q*^*T*^ such that the ’processing rate’ *P* is not less than *p*_*r*_. Noticing that this is a non-linear programming, that is,

$$\begin{array}{l} Minimize\;q^{T}=\sum_{i=1}^{l-1}q_{i}\\ with\;P\left(q_{1},q_{2},\ldots ,q_{l-1}\right)\geq p_{r} \end{array}$$ (5)

Furthermore, the natural extension for the problem presented above is to search the optimal number for each of the holding unit with predefined total number of holding unit which maximizes the ’processing rate’ $P=\sum_{i=1}^{q}\left(1/d_{i}\right)$, *d*_*i*_ denotes the average delay time in each departure route *i*. That is,

$$\begin{array}{l} Maximize\;P\left(q_{1},q_{2},\ldots ,q_{l-1}\right)\\ with\;q^{T}=\sum_{i=1}^{l-1}q_{i} \end{array}$$ (6)

This optimization model can be solved by DP algorithm. All in all, the models given above can effectively integrate the approach routes and departure routes as well as the fixes in the TCA.

## Computational experiments

**Fig 1:**
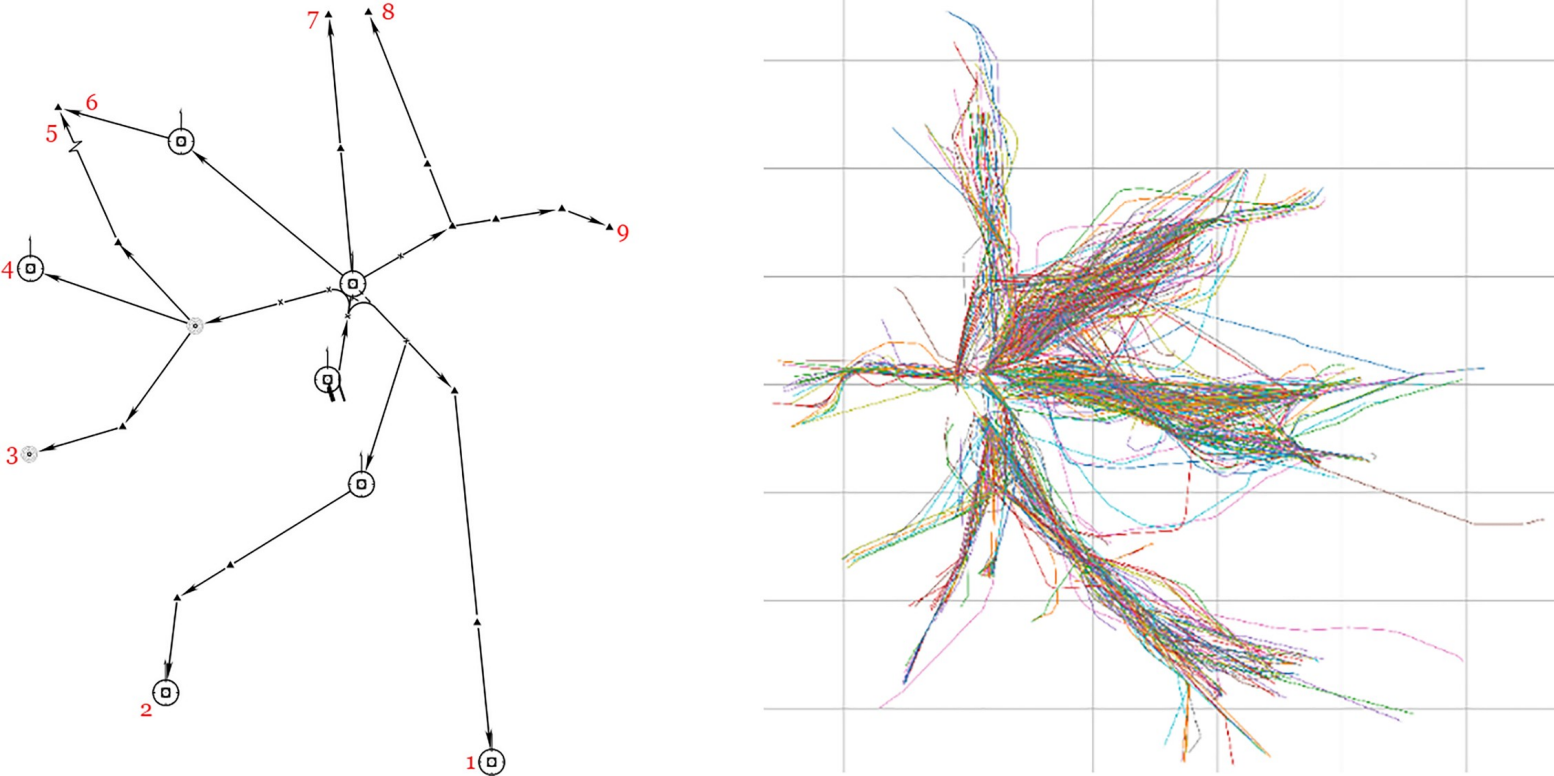
Standard instrument departure chart of the TCA (left) and typical aircraft trajectories (right).

The instances discussed in the following section are derived from actual data for ZABC Airport, as shown in **Fig 1**. A standard instrument departure procedures usually includes a specific number of fixes and the air segments connecting them. Generally, it is very attractive to use real trajectory data, and can capture the specific characteristics of air traffic flow in an airspace in detail. The next section will discuss in detail the various practical situations presented. In addition, a detailed quantitative comparison will also be made between the simulation model and genetic algorithm. In particular, five typical cases have been designed to represent the realistic scenarios.

### Baseline (Case 1)

Firstly, the traffic flow distribution for each departure route is obtained through statistical analysis. In addition, TCA contains 25% of heavy jets and 75% of medium jets. The minimum safety distance is set to 15km, which is quite short. In this context, we calculate key indicators such as the total waiting time of the scheduled flight.

### Case 2

The arrival flow and the departure flow at the fixes are correlated. The ratio of arrival traffic to departure traffic is one. Therefore, there are nine situations. In each case, the traffic arriving at the airport is cleared on average fifteen minutes before departure. The minimum safety distance is set to 20km.

### Case 3

Large safety distance. In this case, we set the minimum safe distance to 25 km. Other parameters are the same as the baseline situation.

### Case 4

High throughput. The throughput of each fix is unlimited as long as the safety distance is guaranteed and other parameters are the same as the baseline situation,.

### Case 5

Normal throughput. The throughput of each fix is set to 0.8* (unlimited throughput), and other parameters are the same as the baseline situation.

In addition, simulation models are applied to evaluate the continuous departure traffic flow of ZABC Airport under high demand. In terms of air traffic distribution, 48% of aircraft take off eastward, 38% take off west and northwest, and about 14% take off southwest and south. In this way, specific simulation scenarios can be generated to obtain simulation results. Prior to the experiment, the basic experimental parameters were fully trained in order to provide a highly realistic simulation environment. As a result, the average interval between the aircraft and the average delay distribution are obtained using the actual training data. Moreover, it should also be noted that the simulations was repeated one hundred times.

Furthermore, our genetic algorithm consists of four components such as selection, crossover, mutation and evaluation. The basic steps of the genetic algorithm are as follows: Firstly, the population is initialized according to the spatial layout of the TCA, and two individuals are selected from the initial population as parents. Then the crossover operation is applied to these two individuals. In order to calculate the fitness value of each individual in the population, our algorithm uses the fitness value of each individual to simulate the configuration of holding units on the departure route. After all the fitness values have been obtained, the algorithm sorts the individuals according to their fitness values and performs iterations.

1. Gene structure: Each gene string corresponds to a holding unit configuration for the departure route, that is, a departure route consists of a specific number of holding units. In particular, the gene string uses integer coding, and the chromosome length is 9.
2. Crossover operation: Crossover operation is an important step to effectively find feasible solutions by exchanging fragments of two gene strings. More specifically, two parents are selected according to their fitness values, and the crossover operation with probability 0.5 is adopted.
3. Mutation operation: Our algorithm mutates an individual with a specified probability. That is, some values in the original chromosome vary randomly with the probability of 0.1.

Table 1 shows the results of various models in terms of the various indicators. The first column reports the scenarios considered. The second column gives the results of the analytical model. Columns 3–4 provide the information of each indicator for simulation model and genetic algorithm. It can be seen from the table that the minimum value of the maximum delay is 362s, which is due to the additional flight time required for airborne holding to meet the minimum interval time between the departure flights. By comparing various indicators, we found that the analytical model outperforms the simulation model and genetic algorithm. In particular, the indicators P1 and P2 corresponding to the analytical model are positively correlated with the other indicators such as P3 and P4. These results are more evident for some scenarios where the average exit delay are more than 28% less when using the analytical model. These differences can be attributed to the following reasons: the arrival time of each aircraft at the initial fix is calculated according to the scheduled processing time for a specified air segment, and due to the optimized configuration of the holding unit, the process time can be optimized for some of the air segments.

**Table 1:** Expected indicators for different models.

	Analytical model	Simulation model	Genetic algorithm
	P1-P2-P3-P4	P1-P2-P3-P4	P1-P2-P3-P4
Case 1	380-135-28-105	660-166-45-146	512-148-36-121
Case 2	371-132-24-104	623-158-38-136	526-142-31-117
Case 3	412-145-48-115	683-181-49-152	641-160-42-143
Case 4	402-141-34-109	644-162-42-141	630-153-38-135
Case 5	362-105-22-90	564-137-37-126	438-128-29-113

[P1: maximum aircraft delay; P2: the average holding time; P3: the number of flights blocked by the sub-segment; P4: average exit delay].

Noticing that the improvements vary from case to case, as shown below. In cases 2 and 5, the flight delays were substantially reduced because the analytical model can release more flights at non-blocking fixes. For the simulation model, since the decision is made at the beginning of the planning period, there may be a tendency to increase the extra flight time in order to reduce flight conflicts. In case 2, the holding unit was cleared 2.1 minutes before the departure fixes on average, which created an opportunity to "release" more aircraft when the airport has cleared but the departure fixes were still blocked. In case 5, the flight arrives later at the departure fixes, which allows the analytical model to configure the optimal number of holding units based on the actual situations. Compared with genetic algorithms, the flight time of flights is reduced by 20% using analytical models. In addition, case 3 and case 4 involve different factors, so there are typical differences between these two models. In case 4, the genetic algorithm has no advantage over the simulation model, because both models cannot clear the departure fixes until the latest possible arrival time of the flight. Finally, the average number of holding unit for the simulation model, genetic algorithm, and analytical model are 13, 10, and 8, respectively.

Compared with the current configuration, Table 2 lists the expected growth percentage of the system throughput that would be attained in each case. By comparing the analytical model with the simulation model, it can be observed that, in most cases, the analytical model can increase the system throughput by 17–22%. These increases result from the reduction of air segment congestion, which can further reduce the airborne holding time. Conversely, when the analytical model is discarded, either because of a very high airborne delay or because the departure fixes are blocked, the results will be poor.

**Table 2:** Expected increase of the throughput compared to the current configurations.

Model type	Analytical model	Simulation model	Genetic algorithm
Case 1	21%	13%	14%
Case 2	17%	10%	11%
Case 3	18%	9%	12%
Case 4	17%	9%	10%
Case 5	22%	13%	16%

After analyzing the performance of the model for different scenarios, the sensitivity of the model to different system parameters can also further analyzed. Next, the potential benefits of the DP model in reducing flight delays were also acquired. Similar to the previous descriptions, we can compare the performance of the DP model and the simulation model in terms of delays for the departure traffic flow. Specifically, three scenarios are considered. In addition, the total numbers of candidate flight levels considered are one-hundred, ninety-three and eighty-five respectively. For the preset number of holding units and "processing time", each row in Table 3 shows the total number of holding units for each departure route.

**Table 3:** Comparisons of the optimization configurations for different methods.

Model type		Number of holding unit for each departure route								
Case 1	Simulation model	15	14	9	11	10	7	11	7	16
	DP model	7	10	12	16	11	14	9	8	13
Case 2	Simulation model	14	16	7	9	12	8	10	10	7
	DP model	10	10	11	14	10	12	8	7	11
Case 3	Simulation model	10	10	9	10	7	9	12	10	8
	DP model	8	9	10	13	8	11	7	8	11

For the DP model, it can be observed that more holding units are allocated to the key air segments which consist of three intersections. In particular, these air segments are very important and more complex than the other departure routes. This may suggest that when more aircraft use these intersecting air segments, it becomes more difficult to manage the departure flow. Finally, the next section will present a comparison of improvements between DP model and simulation model. It can be seen from Table 4 that the benefits associated with DP model are better than that of the simulation model.

**Table 4:** Expected benefits for different models.

DP model	Simulation model
**P1-P2-P3-P4**	**P1-P2-P3-P4**
15%-17%%-14%-15%	10%-9%%-7%-7%
18%-16%%-15%-16%	11%-10%%-9%-10%
14%-20%%-19%-17%	7%-11%%-11%-12%

The key mechanism for the improvement seems to be twofold. First of all, under the premise of ensuring the safety interval, TCA can better respond to the changes in the aircraft number variations and their associated separation requirements. In this way, the dynamic adjustment of the safety interval between aircraft can be realized, thereby improving the utilization rate of the air segment. Secondly, the application of DP modeling technology can improve the allocation number of holding units in the TCA to a certain extent. Finally, a sensitivity analysis is conducted for the optimization model proposed above, which mainly includes the parameters listed below: throughput strategy, TCA configuration, and scheduled flight time.

### (a) Sensitivity analysis with respect to the throughput strategy

In order to evaluate the impact of the throughput strategy on the optimization model for departure flights, two new simulation scenarios were designed based on the baseline scenario by modifying the flight flow statistics rules. One of them is called "throughput-75%". In other words, the 0.75* (unlimited throughput) principle is employed between departure flights. This means that a departure queue less than 0.75* (unrestricted throughput) will be generated according to the estimated take-off time for the flights. The other is called "Throughput-85%". Similarly, The 0.85* (unlimited throughput) principle is employed between departure flights. The results show that relaxing the sequencing strategy between departure flights can reduce delays since the departure flights in the departure queue can be better allocated to holding unit. As a consequence, we observed a reduction of 8% for flight delays. Moreover, it turns out that adopting another strategy did not bring any benefit in reducing delays.

### (b) Sensitivity analysis with respect to the configuration of departure routes

The alternative departure routes in TCA also affect the performance of the optimized model. When a more fine-grained minimum safety distance is applied, the model has more divided sub-segments, thus it can produce a lower level of delays. When assessing the potential benefits of the optimization model, the effect of different TCA configurations on departure flights must be considered. Next, a new simulation scenario is designed based on the baseline scenario, which mainly involves the number of available departure air segments. For departure traffic flow, maintaining a minimum safe distance of 17km will lead to a maximum delay of 393 seconds, while 19 kilometers will result in a maximum delay of 405 seconds. As expected, the results showed that reducing the number of alternative routes would increase delays for departure flights.

### (c) Sensitivity analysis with respect to the flight time

In the tactical phase of departure sequencing, the use of decision support tools is highly dependent on the flight trajectory to determine the estimated time of arrival for each aircraft. According to the optimization model proposed, the longer the flight time of each aircraft, the greater the total travel time. In the baseline scenario, the nominal time is adopted for each aircraft. However, the impact on performance indicators can also be evaluated using different flight times. This difference is due to the fact that when the flight time is increased, it will have a greater impact on the upstream and downstream flights considered in the flight plan, resulting in a delay in the resolution of aircraft conflict. In fact, when the flight time is reduced by 5%, the delay will be reduced by 3%.

## Conclusions

This paper proposes a new method to optimize the configuration of holding units in the TCA. Experimental results show that these optimization models and algorithms help to improve the operating efficiency of TCA. As far as we know, this is the first optimization method that ties flight scheduling to the configuration of holding units for the departure route. The tool developed based on the optimization model proposed in this paper can be applied to the flight scheduling, and thereby determine the best flight sequence. However, it is worth mentioning that further analysis of stochastic factors such as bad weather and rerouting is required in practical applications.

## Supporting information

S1 Data (ZIP)
